# Editorial: Zoonosis associated with parasites and infectious diseases in aquatic animals

**DOI:** 10.3389/fvets.2023.1227007

**Published:** 2023-06-06

**Authors:** Elisabetta Antuofermo, Marta Polinas, Daniele Dessì, Fiona Luisa Henriquez

**Affiliations:** ^1^Department of Veterinary Medicine, University of Sassari, Sassari, Italy; ^2^Department of Biomedical Sciences, University of Sassari, Sassari, Italy; ^3^School of Health and Life Sciences, University of the West of Scotland, Paisley, United Kingdom

**Keywords:** zoonotic disease, fish-borne disease, One Health, bacterial agents, parasitic zoonoses

Zoonoses diseases, playing a central role from the “One Health” perspective, heavily impact both animal and human health due to their interaction in a shared environment (1). The seriousness of zoonotic threats has been gaining momentum in public attention especially after the recent COVID-19 pandemic, and avian flu outbreaks, highlighting the need to implement surveillance of known zoonosis and to address research efforts toward the identification of potential new pathogenic agents, to prevent epidemic consequences (2). To date, over 200 different zoonotic diseases are recognized, representing an important cause of infectious disease in people (3). Zoonoses derived from aquatic animals are increasingly reported in recent years, because of increased undercooked seafood consumption (46%) or infections transmitted by contact between humans and infected animals (20%) (4).

Zoonotic agents from aquatic animals are a heterogenous group of pathogens that includes viruses, bacteria (both Gram-positive and Gram-negative families), fungi, and parasites such as cestodes (e.g., *Diphyllobothrium* spp.), trematodes (e.g., *Opisthorchis* spp.), and nematodes (e.g., *Anisakis* spp.) parasites (4).

Anisakiasis, one of the most frequent fish-borne zoonoses, is caused by the infection of the third-stage larvae of the nematode *Anisakis* sp. after ingestion of undercooked or raw fish (6). Humans act only as an accidental host in the life cycle of anisakid nematodes, and symptoms are mostly related to the gastrointestinal system and allergic reactions (6). This zoonosis is more frequent in, but not limited to, developing countries and rural environments, where food safety practices and surveillance systems are in development. As part of this Research Topic, Liu et al., performed a meta-analysis on the prevalence of anisakid nematodes in fish and reported a mismatch between the high prevalence of *Anisakis* spp. in fish in Chinese regions and the rareness of anisakiasis in humans. This study pointed out the need of implementing the surveillance system as well as making efforts toward raising consumer awareness of threats related to fish-born zoonosis through the consumption of raw fish.

Besides zoonoses of food-borne origin, other routes of transmission of infections need to be examined and some emerging diseases, such as Erysipelas affecting mammals, birds, reptiles, and fish, are triggering increasing concern due to their important risks to public health (5). *Erysipelothrix rhusiopathiae*, a Gram-negative bacterium, is responsible for localized dermatitis or endocarditis and septic arthritis in more severe cases (5). Recently Lee et al., in their contribution to this Research Topic, investigated *E. rhusiopathiae* infecting a free-ranging rough-toothed dolphin (*Steno bredanensis)*. The authors described gross and histopathological findings and characterized isolates, identifying, for the first time, genomic characteristics of the strain involved in a free-ranging dolphin, demonstrating high similarities between this bacterium and those involved in captive dolphin infections. Moreover, they assessed the *in vitro* cytotoxic effects of this pathogen, thus highlighting its great zoonotic potential.

The critical role of the “shared environment” between aquatic animals and humans is also important to consider in a One Health approach, to minimize potential risks to global health. This issue is particularly relevant for marine bivalves, on which climate change can have a deep impact, favoring conditions for zoonotic diseases to spread. Costello et al. in their contributions to this topic, which focused on invasive species introductions in bivalve mollusk as a model group, reported the potential impact of climate changes on parasite invasiveness in their ecosystems. The Authors stated that the modification of water parameters, such as temperature, salinity, acidity, and hypoxia, could deeply impact the host-parasite interaction, by boosting a pathogens' virulence. As an example, the bacterium *Vibrio* species, known for the zoonotic potential of some species, showed increased growth and upregulated virulence genes, during the water temperature rising. In addition, climate change impacts the host's health status increasing its susceptibility because of thermal stress exposure. This alarming condition is reported by other authors that underlined how animals and humans may experience negative effects on their health in altered environmental conditions, leading to a higher vulnerability to infectious diseases (3).

Overall, the contributions to this Research Topic evidenced new aspects of zoonosis of aquatic animals related to parasites and infectious diseases, by investigating variables linked to pathogen molecular characteristics and epidemiology, host susceptibility to pathogenic agents, and the impact of environmental changes on these conditions. These contributions underpin the One Health approach and indicate how providing a deeper understanding of the dynamics beyond the human–animal–environment, and ecosystem interface could help in reducing risks and support prevention and mitigation measures for zoonotic diseases.

## Author contributions

All authors listed have made a substantial, direct, and intellectual contribution to the work and approved it for publication.
